# A Systematic Review Protocol to Identify the Key Benefits and Associated Program Characteristics of Community Gardening for Vulnerable Populations

**DOI:** 10.3390/ijerph17062029

**Published:** 2020-03-19

**Authors:** Danielle Tracey, Tonia Gray, Joanna Sweeting, Jonathan Kingsley, Aisling Bailey, Philip Pettitt

**Affiliations:** 1 Centre for Educational Research, Translational Health Research Institute, Western Sydney University, Sydney, NSW 2751, Australia; 2 Centre for Educational Research, Western Sydney University, Sydney, NSW 2751, Australia; t.gray@westernsydney.edu.au (T.G.); j.sweeting@westernsydney.edu.au (J.S.); 3 School of Health Sciences, Swinburne University of Technology, Melbourne, VIC 3122, Australia; Jkingsley@swin.edu.au; 4 School of Social Sciences, Swinburne University of Technology, Melbourne, VIC 3122, Australia; aabailey@swin.edu.au; 5 Botanic Gardens & Centennial Parklands, Sydney, NSW 2000, Australia; Philip.Pettitt@bgcp.nsw.gov.au

**Keywords:** community gardening, vulnerable, health, well-being, social inclusion, systematic review, protocol

## Abstract

Gardening has long been a popular pastime. There is a growing evidence base for the health and well-being benefits of gardening. Community gardening brings a social aspect to gardening, thereby increasing the potential benefits to include addressing social inclusion and poor community health through sharing of values, support of others, and building networks. This systematic review protocol aims to determine the characteristics of community gardening that could lead to beneficial outcomes such as connection with the community and development of new skills. Thirteen academic databases will be searched for studies looking at the benefits of community gardening, with a focus on vulnerable populations. Data will be extracted from all studies meeting the inclusion criteria and summarized to provide an overview of the current literature. This systematic review aims to provide a comprehensive investigation into community gardening, its benefits, and how they are achieved for the target population. By gathering and synthesizing this information, the review should allow policy makers and practitioners to work more effectively to address health and social inequities, by highlighting areas of need and enabling optimization of future interventions.

## 1. Introduction

Gardening has been a popular pastime globally throughout time and is one of the most commonly accessible ways of experiencing nature [1]. The evidence base for the health and well-being benefits of gardening is rapidly growing with studies showing that community gardening is associated with improvements in symptoms of mental health conditions such as depression and stress [2,3], and in encouraging healthy lifestyle behaviors such as increased fruit and vegetable intake which reduces the risk of cardiovascular disease [4,5,6]. Additionally, gardening has been shown to be beneficial to specific populations, such as older adults in terms of overall health, quality of life, improved cognitive ability, and social benefits [7]. Popularized at times of food shortages during significant events such as the Great Depression and the World Wars, community gardening is viewed as a more recent phenomenon [8]. Community gardens have been seen not only as a source of food produce but also, due to the “community” aspect, a way to address modern day concerns such as social isolation and poor community health, in addition to poor physical and mental health [9,10]. In particular, participation in community gardening has been shown to provide an opportunity for increasing social cohesion through sharing of common aims and values [11], social support through the presence of a community to provide care and comfort in times of crisis [12], and social connection through the development of networks [10,13,14].

To date, systematic reviews in the field of community gardening have focused on food and nutrition related outcomes [6], health and well-being [15], and nutrition and physical health [16]. Three systematic review protocols examining aspects of community gardens have also been published including the prevention of overweight and obesity [17], health and well-being impacts [18], and identification of qualitative and quantitative measures of community gardening [9]. A recent scoping review examined the impact of community gardening on well-being, including physical and mental health, in vulnerable populations [19]. However, their review did not focus solely on vulnerable populations with some included studies examining general populations inclusive of vulnerable individuals. Additionally, their review did not claim to be a systematic review and the search was conducted in 2017, since which time more empirical studies have been published. To our knowledge, no systematic review has been published with a broad focus on all outcomes related to the benefits of community gardening for vulnerable populations specifically. Our landmark systematic review will highlight areas of positive change for participants and program characteristics associated with such change, and thereby provide a solid basis for policy makers and practitioners to work more effectively to address health and social inequities.

### 1.1. Conceptualization of Vulnerability and Community Gardening

#### 1.1.1. Vulnerability

The World Health Organization (WHO) defines vulnerable, disadvantaged, or marginalized groups as those that, “due to factors usually considered outside of their control, do not have the same opportunities as other, more fortunate groups in society” [20]. Collectively, these factors can be related to living in remote or rural areas, low-income or socioeconomic status, being a migrant, refugee or other racial/ethnic minority, Aboriginal or Torres Strait Islander, gender, sexuality, disability, homelessness, and employment status [20,21,22,23]. These characteristics are also referred to as “social determinants” and research to date suggests they are responsible for many inequalities in health across the globe [24,25,26]. Those who are deemed to be vulnerable due to one or more of these social determinants are also more at risk of social exclusion and adverse health outcomes, be it due to a lack of housing, education, transport, or other resources that others in society have access to [24]. Therefore, it is imperative that vulnerable populations are a focus of health research and programs designed to enable participation of at-risk individuals, rather than widening health inequalities through inaccessible interventions [27]. This paper adopts the term vulnerable to denote this population, while acknowledging the range of synonyms available in the literature.

Community gardening programs, and their founding organizations are now seeking to deliver community gardening to low income communities as a means of addressing the health, social, and economic adversity encountered in these communities. For example, the Royal Botanic Gardens and Domain Trust (RBG&DT) Community Greening Program in New South Wales, Australia [28] was designed to serve those in social housing communities, i.e., those of low-income or socioeconomic status, the unemployed, or people with a disability who are at greater risk of health inequality and social exclusion. There are currently numerous sites across New South Wales, Australia where community gardening programs have been initiated and are being maintained (see: https://tinyurl.com/yxkl2clp) [29]. Similarly, The London Food Poverty Project, in Southwark, aims to foster resilience and knowledge in low income communities so members feel confident to mediate the triggers of food poverty. It achieves this by building a sense of community where people can learn about growing, cooking, and the consumption of healthy food [30].

#### 1.1.2. Community Gardening

The term “community gardening” has been used in the literature to describe a range of gardening type activities from small neighborhood gardens to larger allotment gardens of up to 1000 m² [15]. There is currently no consensus regarding the definition of community gardening in the literature [31]. However, there is a common thread to community gardening terminology which combines the concept of a public setting, be it in a local neighborhood, hospital, or school [18,32] with the act of growing food (which can include fruit, vegetables, and/or livestock) or flowers in a collaborative, communal, and cooperative way for the benefit of all involved [9,15,32]. Allotment gardening, by contrast, occurs where land is acquired via a lease or rent for personal use [9]. Nonetheless, where allotment gardening meets the criteria of growing food or flowers in a communal way to benefit participants, it too can be considered as community gardening. Similarly, community gardening can occur within an urban garden context, however, it is distinguished by its communal element.

A recent review of international literature found community gardens are generally governed in one of six forms, grouped into top-down or bottom-up approaches [33]. A purely top-down approach comprised the use of private land under the governance of a Trust with little to no community support, where as a top-down approach incorporated community help [33]. Overall, bottom-up approaches were more common with gardens instigated and maintained by members of local housing estates or other community groups, with varying levels of support from professionals (either in a paid, volunteer, or administrative capacity) [33].

The benefits of community gardening are numerous, building upon the previously mentioned physical and mental health benefits of gardening in general. Due to the grounding in a community setting, community gardens have been shown to improve connections within the local community, thereby reducing social isolation [34,35]. Additional benefits attributed to participation in community gardens include promoting a healthy diet through increasing fruit and vegetable intake [5], building a sense of identity and ownership [35], stress relief [35], encouraging contact with nature [1], and increasing social capital [36,37].

Empirical studies have highlighted the salience of these benefits for vulnerable populations with community gardens associated with positive impacts for refugee populations [38] and those in lower socioeconomic areas including housing estates [39]. Community gardens have also been shown to help migrants create a sense of belonging in their new home and in some cases, facilitate connection with the local community [40]. Additionally, a study in Melbourne, Australia has shown that community gardens are valued not only for sociocultural benefits and as a place of community interaction for both Australian and foreign born non-European gardeners, but also as a source of food production [41]. For vulnerable communities where income is low, the benefit of access to nutritious food, and subsequent food security and physical health, is featured as a key benefit of community gardening.

A recent qualitative evaluation of The London Food Poverty Project [30] for low income communities identified community gardening as a conduit for connecting people, building confidence, well-being, and improving both gardening and cooking skills. In a unique mixed-method study, participants of RBG&DT’s Community Greening Program reported a significant increase in their shared emotional connection with the community as measured by a quantitative survey at the beginning of their participation and six to seven months later [28]. Participants who were not eating any fruit and vegetables or cooking healthy food upon commencing at the community garden had indeed changed these behaviors at the post test. No other significant positive changes were reported, although qualitative data also revealed positive changes in both inter- and intrapersonal outcomes.

Although evidence of the health benefits of community gardening is mounting, a broader range of social outcomes that could be particularly valued for vulnerable populations, such as skill and knowledge acquisition and participation in training and employment, remain under researched. Indeed, the Botanic Gardens Conservation International [42] network laments that one of the key factors limiting botanic gardens from taking a greater role in addressing issues of social equality is “the paucity of evidence demonstrating its impact”.

This systematic review will aim to determine the benefits of community gardening for vulnerable populations, and the characteristics of community gardens that lead to beneficial outcomes including, but not limited to:

1. Food and nutrition security;
2. Connections with the community that the gardens enhance;
3. Ownership and care for the environment through participation in this activity;
4. Use and enjoyment of social housing green space;
5. Development of skills, knowledge and capacity; participation in education, training, and employment;
6. Physical and mental health through this amenity.

There are no age limits applied on the study population as gardening is an intergenerational activity that can be undertaken by individuals of all ages [43]. The review will include all relevant studies that examine outcomes related to active participation (as opposed to simply purchasing produce from a community garden) in a community garden.

## 2. Methods

This systematic review will be conducted in line with the Preferred Reporting Items for Systematic Reviews and Meta-Analyses (PRISMA) guidelines [44].

### 2.1. Searches

This systematic review will include international peer-reviewed literature, published in English. The search will be limited to literature published between 1985 and 2020. This period has been selected so as to capture relevant articles published since the development of the Botanic Gardens Conservation International (BGCI) network. This global network aimed to provide guidance, support, and to empower members and the wider community to enable conservation of plant species for the well-being of people and the planet [45]. Involvement in community gardening is one means by which the BGCI acts to achieve its aim.

Search results will be screened initially by one author with potentially relevant abstracts screened independently by a second author. Academic searches will be conducted using the following databases: (1) PubMed, (2) Medline, (3) Scopus, (4) ScienceDirect, (5) Cumulative Index to Nursing and Allied Health Literature (CINAHL), (6) PsycINFO, (7) Web of Science, (8) Academic Search Complete, (9) Education Source, (10) Education Resources Information Center (ERIC), (11) Psychology and Behavioral Science Collection (12), SocINDEX, and (13) Allied Health and Complimentary Medicine Database (AMED). Forward and backward citation searches will be conducted on all included studies. Grey literature will also be searched through Google Scholar (first 50 hits) and OpenGrey.

### 2.2. Search Strategy and Terms

This protocol was developed by an interdisciplinary team with a diverse range of expertise (including specialization in psychology, education, public health, horticulture, and anthropology) and reflected a collaborative effort to ensure a thorough and systemic search process. Table 1 lists the intended search terms under two headings, “gardening” and “population”, which will be combined in the search process with “AND” with searches conducted on title and keywords. Search results will be managed using the Endnote reference management software.

### 2.3. Article Screening

This review aims to examine outcomes related to collaborative, communal and cooperative “community gardening”, including “allotment gardening” as described in the Introduction. An initial screening process will remove all duplicates, articles not in English, and other studies identified as clearly unsuitable for inclusion, for example, due to a lack of focus on community gardening. Both qualitative and quantitative studies will be included in this review. This will include pre-post, quasi-experimental, randomized controlled trials (RCTs), case-control, cohort, and cross-sectional studies.

#### 2.3.1. Inclusion Criteria

This review will include papers that examine the impact or outcomes of community gardening programs for active participants, i.e., those involved in gardening activities such as maintenance, planting, and harvesting. To be included, the garden projects must involve a social aspect, i.e., conducted within a community setting. The populations of interest will be those identified by the study as vulnerable due to factors such as socioeconomic status, income, employment, ethnicity, and refugee status. All ages and settings (e.g., schools, hospitals, and social housing) will be included.

#### 2.3.2. Exclusion Criteria

Any study not meeting the inclusion criteria will be excluded from the review. Additionally, studies focusing on a population with a specific health condition (e.g., Alzheimer’s, dementia) will be excluded. Although it is acknowledged that there is comorbidity between health conditions and vulnerability, the primary purpose of the systematic review is to evaluate empirical studies focused on vulnerable populations, as described in Table 1, rather than on individuals with specific medical conditions. Only original research will be included, and therefore literature reviews, commentary/opinion/perspective articles, and theoretical papers will be excluded. A list of all excluded studies will be available in tabulated form with reasons for exclusion.

### 2.4. Article Evaluation

Following the initial screening process, all remaining studies will be assessed for inclusion by two authors independently, based on title and abstract. The full text of all studies potentially meeting the inclusion criteria will then be accessed and assessed by two authors, independently. Any disagreements will be resolved through discussion, or where necessary, deferral to a third author.

### 2.5. Data Extraction

Data will be extracted from the included studies into a bespoke spreadsheet developed for this study. Extracted data will include author, setting/country, year of publication, funding sources, aim, population (including demographics such as mean age and range, gender, racial background, nature of vulnerability), study type, intervention characteristics (e.g., length, number of sessions, number of participants, personnel required), outcomes measured, and key findings.

### 2.6. Data Synthesis

Due to the expected diverse nature of the identified studies and their outcomes, this systematic review will not attempt any meta-analyses but rather present a narrative synthesis of the included papers, that is, statistical analyses will not be used to combine the data derived from the systematic review. The narrative synthesis will assess the outcomes measured and the key findings across each outcome. Additionally, these findings will be evaluated against study type and intervention characteristics.

### 2.7. Quality Assessment

Quality of the included studies will be assessed using the Trisha Greenhalgh hierarchy [46], given the expected diversity of study type, i.e., both quantitative and qualitative studies. The Greenhalgh hierarchy assesses five questions including:

1. Was the study original?
2. Who was the study about?
3. Was the study design sensible?
4. Was systematic bias avoided or minimized?
5. Was the study large enough, and long enough, to make the results credible?

The quality assessment will be conducted independently by two authors.

## 3. Limitations

This systematic review will be limited to articles in English due to pragmatic reasons, and therefore could miss relevant papers published in other languages.

## 4. Conclusions

The proceeding systematic review will provide a comprehensive investigation into community gardening and its benefits specifically for vulnerable populations. It is anticipated that this review would add further evidence to the health benefits of community gardening, and advance current knowledge by extending the type of outcomes considered in the research. Furthermore, it will include commentary on the characteristics of community gardening that lead to these benefits. The results of this review should help inform the direction of further studies and community programs, including optimization of interventions, by highlighting areas in which research is lacking and program characteristics which have the most potential for greatest benefit to participants. By gathering and synthesizing this information it should help policy makers and practitioners work more effectively with vulnerable populations. In turn, this should allow community gardening to better meet the target group’s needs and address health and social inequalities via accessible and community-based programs.

## Figures and Tables

**Table 1:** Proposed search terms.

Gardening	Population	
Community Garden *	Vulnerable	Public housing
Community Greening	Disadvantag *	Poverty
Allotment Garden *	Marginal *	Inequalit *
Garden *	Refugee	Aboriginal
Greening	Social housing	Disabil *
Urban garden * Urban farm Urban agriculture	Socio * economic	Unemploy *
	Low-income	Homeless *
	Minority	Migrant
	Indigenous	

* indicates wild card inserted.
